# CDC42 Might Be a Molecular Signature of DWI-FLAIR Mismatch in a Nonhuman Primate Stroke Model

**DOI:** 10.3390/brainsci13020287

**Published:** 2023-02-08

**Authors:** Haiping Huang, Shuang Wu, Chengwei Liang, Chao Qin, Ziming Ye, Jingqun Tang, Xiangren Chen, Xiaoyun Xie, Cilan Wang, Jinfeng Fu, Mengyu Deng, Jingli Liu

**Affiliations:** Department of Neurology, The First Affiliated Hospital of Guangxi Medical University, Nanning 530021, China

**Keywords:** ischemic stroke, DWI-FLAIR mismatch, CDC42, RHOA, nonhuman primate

## Abstract

No definitive blood markers of DWI-FLAIR mismatch, a pivotal indicator of salvageable ischemic penumbra brain tissue, are known. We previously reported that CDC42 and RHOA are associated with the ischemic penumbra. Here, we investigated whether plasma CDC42 and RHOA are surrogate markers of DWI-FLAIR mismatch. Sixteen cynomolgus macaques (3 as controls and 13 for the stroke model) were included. Guided by digital subtraction angiography (DSA), a middle cerebral artery occlusion (MCAO) model was established by occluding the middle cerebral artery (MCA) with a balloon. MRI and neurological deficit scoring were performed to evaluate postinfarction changes. Plasma CDC42 and RHOA levels were measured by enzyme-linked immunosorbent assay (ELISA). The stroke model was successfully established in eight monkeys. Based on postinfarction MRI images, experimental animals were divided into a FLAIR (−) group (*N* = 4) and a FLAIR (+) group (*N* = 4). Plasma CDC42 in the FLAIR (−) group showed a significant decrease compared with that in the FLAIR (+) group (*p* < 0.05). No statistically significant difference was observed for plasma RHOA. The FLAIR (−) group showed a milder neurological function deficit and a smaller infarct volume than the FLAIR (+) group (*p* < 0.05). Therefore, plasma CDC42 might be a new surrogate marker for DWI-FLAIR mismatch.

## 1. Introduction

Salvaging the ischemic penumbra is the primary purpose in the acute phase of ischemic stroke treatment but is restricted by a transient time window. The mismatch between diffusion-weighted MRI (DWI) and fluid-attenuated inversion recovery (FLAIR) MRI has served as an indicator of potentially salvageable ischemic cerebral tissue for unclear onset stroke, as validated by clinical trials [1,2]. The DWI-FLAIR mismatch can be applied to screen ischemic penumbra imaging in patients with acute ischemic stroke on awakening from sleep or unknown onset time for intravenous thrombolysis, as recommended by both the guidelines of 2019 AHA/ASA and 2021 ESO for acute ischemic stroke [3,4]. DWI-FLAIR mismatch can be defined as the visual presence of a parenchymal ischemic lesion hyperintensity occurring on DWI but not on FLAIR [5]. This mismatch between imaging methods is based on the time sensitivity of edema development, because cytotoxic edema promptly becomes visible on DWI within a few minutes after stroke onset, while vasogenic edema appears several hours later on FLAIR [6,7]. However, the underlying pathophysiological mechanisms of DWI-FLAIR mismatch, including plasma biomarkers, remain unclear. Moreover, DWI-FLAIR mismatch is subjective based on visual judgment [1] and depends on specialized medical equipment. Blood biomarkers are accessible, economical, and patient-friendly diagnostic tools that are easily understood by clinicians [8]. Therefore, we propose a more objective and convenient biomarker to describe DWI-FLAIR mismatch.

Emerging blood-derived biomarkers reflect the diagnosis, severity, and prognosis of stroke [9] but rarely focus on the penumbra. Our previous studies confirmed that ras homolog family member A (RHOA) and cell division cycle 42 (CDC42) might be useful for the early evaluation of ischemic penumbra and could serve as potential serum biomarkers in a rat focal cerebral ischemia model [10]. Moreover, a sequential transcriptome analysis of the penumbra after ischemic stroke identified RHOA expression downregulation in the ipsilateral motor cortex [11], further supporting the viewpoint that RHOA could act as a biomarker for the penumbra. CDC42 and RHOA belong to the Rho family of low molecular weight (∼21 kDa) guanine nucleotide binding proteins and are involved in critical cellular processes, including neuronal morphology, axon guidance, and neuronal survival and death [12]. Moreover, CDC42 and RHOA serve as key modulators in the cascade of ischemic stroke involving apoptosis, excitotoxicity, platelet function, neuroinflammation, and blood–brain barrier (BBB) balance [13,14,15,16], emphasizing CDC42 and RHOA as key endogenous mediators in the penumbra. However, few studies have focused on the relationship between these blood biomarkers and neuroimaging during the hyperacute phase of ischemic stroke, especially in nonhuman primate.

DWI-FLAIR mismatch has been adopted in clinical practice, but its related body fluid markers have received little attention. In the current study, we evaluated whether the levels of CDC42 and RHOA in plasma are related to DWI-FLAIR mismatch in the hyperacute phase of stroke in nonhuman primates. This finding will provide new insights into a more objective assessment of the presence of salvageable ischemic penumbra brain tissue to ensure maximum benefit from reperfusion therapy.

## 2. Materials and Methods

### 2.1. Animals

A total of 16 healthy male cynomolgus macaques (9–13 years old, weighing 8–13 kg) were purchased from Lingkang Senoco Biotechnology Company (Nanning, China). All experiments were approved by The Animal Care and Welfare Committee of Lingkang Senoco Biotechnology Company.

All animals were housed individually indoors under standard conditions (12:12 h light:dark, light on from 07:00 to 19:00, a constant room temperature of 20–24 °C, and humidity at 60%). The laboratory diet was provided two times daily, supplemented with fresh fruit and vegetables and drinking water. The animals were trained in memory function 14 days before surgery.

### 2.2. Methods

#### 2.2.1. Middle Cerebral Artery Occlusion (MCAO) Ischemic Stroke Model

Before the operation, each cynomolgus macaque was fasted for 24 h and deprived of water for 6 h. Anesthesia was induced by intramuscular injection of ketamine (0.6–0.8 mL) and maintained with isoflurane inhalation (2%) mixed oxygen (O_2_ flow rate of 2 L/min) throughout the operation. Atropine sulfate (0.3 mL) was used to reduce glandular secretion. Monitoring equipment was used to measure physical parameters, including blood pressure, respiratory frequency, oxygen saturation, electrocardiogram, and rectal temperature. The vital sign parameters of the animals were monitored and maintained within a normal range throughout the experiment.

**Figure 1:**
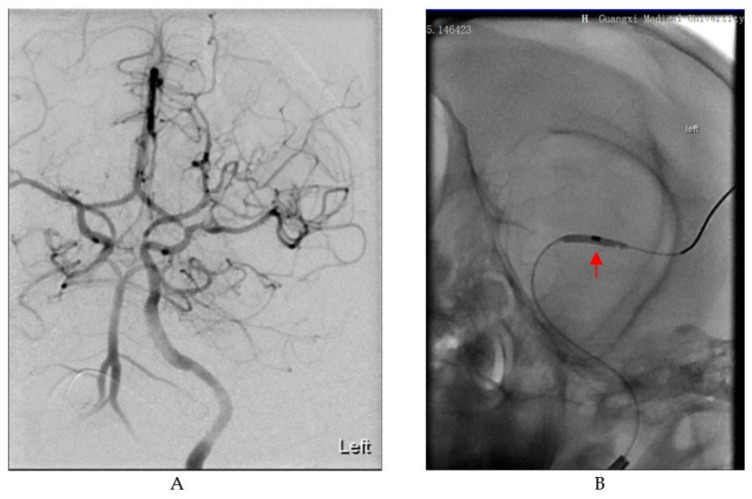
Digital subtraction angiography (DSA) images in the process of establishing the cynomolgus monkey stroke model. (**A**) Image of cerebral vascular anatomy in cynomolgus monkeys under DSA. (**B**) A balloon dilates in the M1 segment of the MCA as indicated by the red arrow.

In the supine position, the animals were immobilized on the operating table. Endovascular ischemic stroke induction was performed by two skilled neurointerventional specialists. As we previously described [17], a 6 French sheath was inserted through the femoral artery. Under the guidance of a super-slippery guide wire, cerebral angiography was performed to verify catheter placement and to assess blood flow status. Imaging showed a well-developed left middle cerebral artery (MCA) with smooth blood flow (Figure 1A). Then, under the guidance of the catheter, the microcatheter (Excelsior^®^ SL-10, Stryker, Kalamazoo, MI, USA) and microguide wire (Synchro^®^-14, Stryker, Kalamazoo, MI, USA) were advanced to the distal end of the M1 segment of the left middle cerebral artery. Next, the balloon (1.5 mm × 9 mm, gateway) was delivered through the microguide wire to the left MCA M1 segment and expanded to block blood flow (Figure 1B). Cerebral angiography was employed to confirm that the balloon completely occluded the MCA. The balloon was withdrawn after 75 min of occlusion. Subsequently, another angiography was performed to evaluate the recanalization of the blood vessels. The interventional devices were removed after the operation, the puncture point was compressed for 30 min, and sterile bandages were applied. Then, an MRI scan was performed on each cynomolgus macaque. Every effort was made to prevent suffering and pain in the animals. Intensive postoperative care was provided until the animals could feed themselves.

#### 2.2.2. MRI Scanning

MRI scanning was performed on a 3 T Siemens Trio scanner (Siemens, Berlin, Germany) with a 32-channel head coil in the radiology department of the First Affiliated Hospital of Guangxi Medical University. MRI, DWI, and MRA sequences were recorded. The animals were kept under anesthesia (isoflurane 2%, O_2_ flow rate of 2 L/min) during scanning. Before the operation, health examinations, including brain MRI, were executed for all animals to rule out neurological dysfunction and cerebral vascular and intracranial lesions. Following the operation, MRI was performed again to confirm successful modeling and assess infarct lesions.

#### 2.2.3. MRI Analysis

The raw DICOM data acquired from the MRI scan sequences were transferred to the GE postprocessing workstation AW VolumeShare 4.7 for subsequent analysis and processing. The infarct volume was measured on the DWI sequences. DWI-FLAIR mismatch was defined as the presence of parenchymal hyperintensity on DWI but not on FLAIR, labeled as the FLAIR (−). The FLAIR (+) was defined as the presence of parenchymal hyperintensity on both DWI and FLAIR. All imaging analyses were performed by two independent experienced neurologists.

#### 2.2.4. Neurological Assessment

Neurological assessment was performed 24 h after the induction of stroke, in order to avoid the effects of anesthesia on neurological function. The standardized neurological function scores of each cynomolgus macaque was assessed by two independent researchers with the Non-Human Primate Neurological Scale (NHPNS) [18]. This is a 100-point scale, with 28 points for consciousness, 22 points for sensory system, 32 points for motor system, and 18 points for skeletal muscle coordination. The higher the score, the more serious the neurological deficit.

#### 2.2.5. Plasma Collection

Two milliliters of peripheral venous blood was collected from 3 healthy control monkeys after MRI and neurological evaluation. Approximately 2 mL of venous blood was obtained from each experimental interventional monkey following the completion of the MRI and confirmation of the successful MCAO model by MRI. Blood samples were anticoagulated with EDTA. The blood samples were centrifuged within 60 min of collection at 3000 rpm at 4 °C for 15 min. Plasma samples were aliquoted and stored at −80 °C until further analysis.

#### 2.2.6. Enzyme-Linked Immunosorbent Assay (ELISA)

The total CDC42 and RHOA levels in plasma were measured using commercial ELISA kits according to the manufacturer’s instructions (MyBiosource, San Diego, CA, USA). The steps of the experimental process were strictly in accordance with the manufacturer’s instructions. The means of the duplicate experiments were used for the statistical analysis. Investigators were blinded to information about the samples.

#### 2.2.7. Statistical Analysis

Measurement data are expressed as the mean ± SD. Independent-samples *t*-test and one-way ANOVA were used to compare continuous variables. Data analyses were performed with GraphPad Prism (version 9.3, GraphPad Software, San Diego, CA, USA). The significance level for all analyses was set at *p* < 0.05.

## 3. Results

### 3.1. General Results of the Stroke Model and MRI Assessment

**Figure 2:**
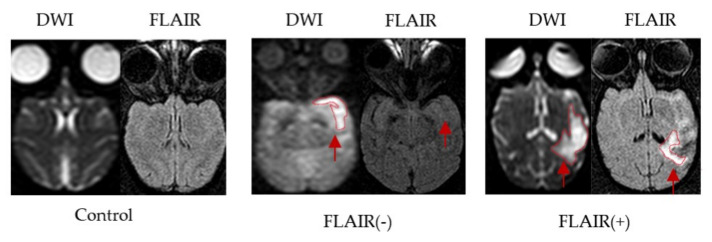
Brain MRI images of the MCAO model in cynomolgus macaques. Representative images of infarction lesions on DWI and FLAIR in cynomolgus macaque brains after MCAO (red arrow).

The MRI and neurological examination of three healthy control monkeys were normal. The total experiment time from MCAO to MRI completion and blood collection was approximately 2 h. Successful establishment of the stroke model was confirmed by brain parenchyma hyperintensity on DWI and the presence of neurological deficits. The stroke model was successfully established in 8 of the 13 monkeys and failed in 5 monkeys. Two monkeys died of cerebral herniation, one died of intestinal obstruction and infection, and two monkeys had no neurological deficits and no infarcts on MRI. Based on the FLAIR signal on MRI findings, eight postoperative cynomolgus macaques for the MACO model were divided into a FLAIR (−) group (*N* = 4) and a FLAIR (+) group (*N* = 4) (Figure 2).

### 3.2. Milder Neurological Deficits and Smaller Infarct Volumes in the FLAIR (−) Group

**Figure 3:**
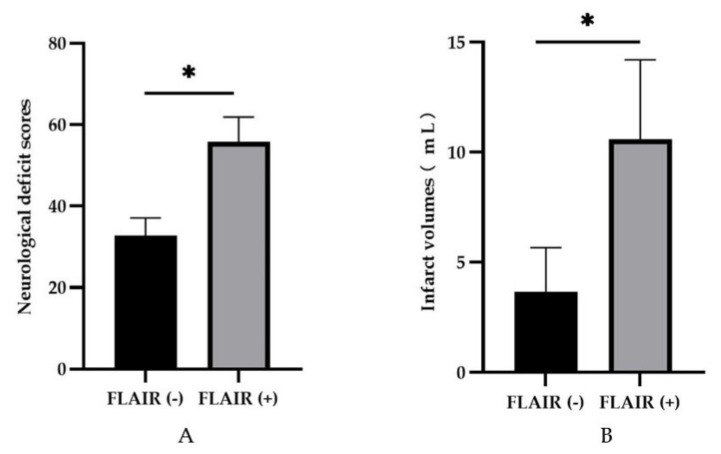
The FLAIR (−) group had lower neurological deficit scores and smaller infarct volumes than the FLAIR (+) group. * Presented *p* < 0.05. (**A**) The postoperative neurological deficit scores of the monkeys in each group 24 h after infarction. (**B**) The infarct volumes on DWI in each group.

Varying degrees of paralysis developed in each of the postoperative cynomolgus monkeys. Neurological scores showed significant differences between the two postoperative groups (*p* < 0.05, Figure 3A). The lower the score, the milder functional outcome. Therefore, there was less neurological impairment in the FLAIR (−) group. To assess the extent of brain tissue damage in each group, we measured the infarct volumes. Smaller infarct volumes were observed in the FLAIR (−) group than in the FLAIR (+) group (*p* < 0.05, Figure 3B).

### 3.3. CDC42 and RHOA Plasma Levels

**Figure 4:**
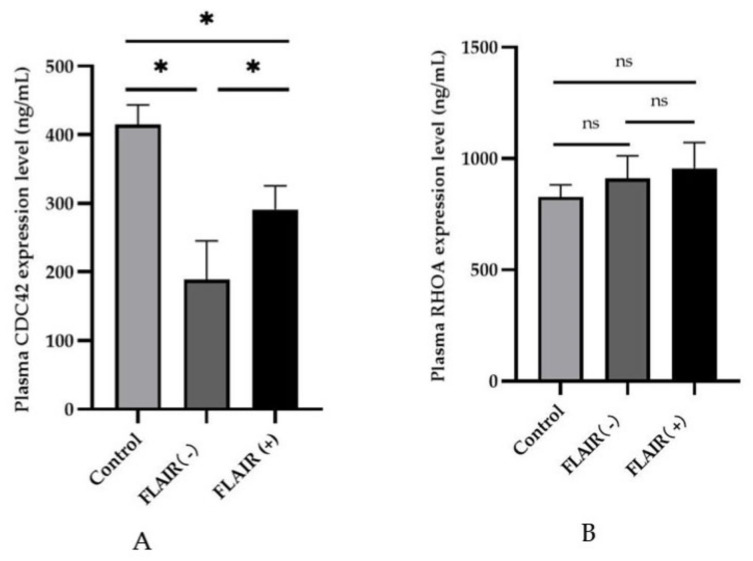
Plasma CDC42 and RHOA expression levels in the control, FLAIR (−), and FLAIR (+) groups. (**A**) Plasma CDC42 expression in each group. * Presented *p* < 0.05. (**B**) Plasma RHOA expression in each group. ns, *p* > 0.05.

In plasma, the expression of CDC42 in the FLAIR (−) group and FLAIR (+) group was lower than that in the control group, and both differences were statistically significant (*p* < 0.05, Figure 4A). Moreover, the expression of plasma CDC42 was lower in the FLAIR (−) group than in the FLAIR (+) group (*p* < 0.05). However, RHOA expression in plasma did not change significantly among the control, FLAIR (−) group, and FLAIR (+) group (*p* > 0.05, Figure 4B).

## 4. Discussion

Our results showed that plasma CDC42 levels were lower in the hyperacute phase of stroke than in the control group. The FLAIR (−) group had a more significant decline in CDC42 plasma levels than the FLAIR (+) group. Plasma CDC42 levels might be related to DWI-FLAIR mismatch. Smaller infarct volumes and milder neurological deficits were observed in the FLAIR (−) group than in the FLAIR (+) group. These results suggest that CDC42 is a potential molecular signature of DWI-FLAIR mismatch, and lower CDC42 levels indicates a better prognosis of stroke in our study. However, no statistical significance was observed between RHOA levels in plasma and imaging markers. To the best of our knowledge, this is the first study to focus on the relationship between plasma CDC42 levels and DWI-FLAIR mismatch, especially in a nonhuman primate MCAO model. In the current study, the plasma CDC42 level is associated with DWI-FLAIR mismatch, suggesting that CDC42 may be a potential surrogate marker for identifying the presence of ischemic penumbra.

Reperfusion therapy based on DWI-FLAIR mismatch could rescue ischemic penumbra brain tissue in different types of stroke, including infratentorial stroke and lacunar infarction [19,20,21]. However, the DWI-FLAIR mismatch method may incorrectly assess some potential thrombolysis patients identified by PWI-DWI mismatch, despite FLAIR hyperintensity [22]. In addition, the MRI mismatch results may be affected by parameter settings and clinician reading [23,24]. Moreover, clinical applications of MRI are primarily limited to major academic institutions because MRI is not a routine examination in emergency departments. Therefore, a surrogate marker for DWI-FLAIR mismatch for clinical convenience and objectivity is urgently needed.

Studies correlating molecular biomarkers and neuroimaging mismatches in acute stroke have been limited. A recent study of rats within 4.5 h after MCAO focused on the pathological substrates correlated with DWI-T2 mismatch showed that CARMIL3 expression in neurons was significantly upregulated in the mismatch area, which was associated with neuronal edema, impaired autophagy, and oxidative stress [25]. Another study found an association between the hyperacute plasma levels of F2-isoP and ORAC and PWI-DWI mismatch, characterized by oxidative stress-induced molecules in the ischemic penumbra of patients with acute ischemic stroke [26]. In our study, we used well-accepted radiographic DWI-FLAIR mismatch guidelines to evaluate the relationship between plasma CDC42 levels and DWI-FLAIR mismatch.

Previous research confirmed that reduced levels of CDC42 protein are associated with acute ischemic stroke [27,28]. These results were consistent with our findings that showed decreased CDC42 levels in the stroke model groups. Furthermore, the CDC42 level decreased more significantly in the DWI-FLAIR mismatch subgroup compared to the DWI-FLAIR matched group. Additionally, reduced CDC42 levels were found in coronary heart disease patients compared to controls, indicating inflammation [29]. Downregulation of CDC42 attenuates neuronal apoptosis in ischemic stroke [14,30]. Moreover, CDC42 signaling is associated with vascular inflammation and the progression of atherosclerosis, which may further contribute to the development of stroke [15]. However, no investigation has shown an association between CDC42 and DWI-FLAIR mismatch in ischemic stroke. This study found a novel association between CDC42 levels in plasma and DWI-FLAIR mismatch.

A decrease in CDC42 plasma levels was observed in the DWI-FLAIR mismatch group, suggesting that this decrease may be a response to ischemic injury. The inflammatory phase occurs almost immediately following cerebral ischemic injury, resulting in further damage to cells. Neutrophils are greatly affected by ischemia and migrate to the cerebral parenchyma within minutes of ischemic injury [31]. Moreover, neuroinflammation progressively damages the penumbral tissue [32,33]. Platelets are major mediators of thrombotic inflammation and contribute to reperfusion injury [34]. However, the inhibition of CDC42 significantly decreases neutrophil polarity and chemotaxis [35,36]. Because it contributes to the inhibition of the inflammatory process of neutrophils, a reduction in CDC42 in the DWI-FLIAR mismatch group may delay penumbra tissue damage from inflammation. More importantly, the downregulation of CDC42 inhibits platelet activation and thrombus formation [37,38], which may decrease the risk of thrombosis and alleviate ischemia–reperfusion injury. Consistent with the documented results, CDC42 plasma levels were reduced in the mismatch group and played a crucial protective role in delaying neuronal damage.

CDC42 and RHOA play important roles in neuronal remodeling and polarization [39]. In a mouse stroke model, CDC42, not RHOA, was the main protein related to cytoskeleton formation and cell polarization [40]. Downregulated expression of CDC42, but not RHOA, markedly attenuated cell chemotaxis via regulation of motility [41]. These results strongly suggest that CDC42, rather than RHOA, is vital for cell polarization and chemotaxis, which may partly explain the lack of statistical significance between RHOA and DWI-FLAIR mismatch. Furthermore, the expression level of RHOA in the blood of patients with acute ischemic stroke is affected by gender and the time of the examination [42]. Peripheral blood RHOA is specifically expressed in males with acute stroke [42]. All of the cynomolgus macaques in our experiment were male, which may have led to no statistical difference in RHOA. As peripheral blood markers of the ischemic penumbra, the expression patterns of CDC42 and RHOA in nonhuman primate stroke models differ from those in rats that we previously reported [10]. A possible explanation for this discrepancy is species diversity.

Some limitations are associated with this study. First, the experimental sample size was relatively small, limited by the rarity of nonhuman primates and the difficulty of the endovascular stroke model. Second, we were not able to continuously monitor the relationship between the expression level of CDC42 and RHOA, neurological progression, and MRI images. The main reason for this was that repeated anesthesia within a short period of time resulted in a high mortality rate in the monkeys, because anesthesia was required for every blood collection and MRI examination. Third, we did not pay attention to collateral circulation, which may affect FLAIR signals and ischemic penumbras. Fourth, the results of this experiment have not been verified in clinical patients.

## 5. Conclusions

Downregulation of plasma CDC42 levels, a smaller infarct volume, and fewer neurological deficits were observed in the DWI-FLAIR mismatch group. As a result of these findings, we propose that CDC42 may be a new surrogate marker for DWI-FLAIR mismatch that could provide a convenient, accurate, and economical basis for the clinical diagnosis of salvageable ischemic penumbra brain tissue.

## Data Availability

Not applicable.
